# Research on the Multi-Element Synthetic Aperture Focusing Technique in Breast Ultrasound Imaging, Based on the Ring Array

**DOI:** 10.3390/mi13101753

**Published:** 2022-10-16

**Authors:** Yang Wu, Wendong Zhang, Xingling Shao, Yuhua Yang, Tian Zhang, Miao Lei, Zhihao Wang, Bizhen Gao, Shumin Hu

**Affiliations:** 1 State Key Laboratory of Dynamic Measurement Technology, North University of China, Taiyuan 030051, China; 2 National Key Laboratory for Electronic Measurement Technology, School of Instrument and Electronics, North University of China, Taiyuan 030051, China

**Keywords:** ultrasonography, breast imaging, ring array, synthetic aperture, multi-element synthetic aperture focusing

## Abstract

As a widely clinical detection method, ultrasonography (US) has been applied to the diagnosis of breast cancer. In this paper, the multi-element synthetic aperture focusing (M-SAF) is applied to the ring array of breast ultrasonography (US) imaging, which addresses the problem of low imaging quality due to the single active element for each emission and the reception in the synthetic aperture focusing. In order to determine the optimal sub-aperture size, the formula is derived for calculating the internal sound pressure of the ring array with a 200 mm diameter, and the sound pressure distribution is analyzed. The ring array with 1024 elements (1024 ring array) is established in COMSOL Multiphysics 5.6, and the optimal sub-aperture size is 16 elements, according to the sound field beam simulation and the directivity research. Based on the existing experimental conditions, the ring array with 256 elements (256 ring array) is simulated and verified by experiments. The simulation has a spatial resolution evaluation in the k-Wave toolbox, and the experiment uses nylon rope and breast model imaging. The results show that if the sub-aperture size has four elements, the imaging quality is the highest. Specifically, the spatial resolution is the best, and the sound pressure amplitude and signal-to-noise ratio (SNR) are maintained at a high level in the reconstructed image. The optimal sub-aperture theory is verified by the two kinds of ring arrays, which also provide a theoretical basis for the application of the multi-element synthetic aperture focusing technology (M-SAF) in ring arrays.

## 1. Introduction

Breast cancer, as one of the most common malignancies in worldwide, is extremely harmful to women [1]. In recent years, the incidence of breast cancer has been increasing and patients are getting younger, which has become a major public health problem. By 2020, there were 2.3 million new cases [2]. In 2020, the number of breast cancer deaths worldwide was as high as 685,000. An early diagnosis and prompt treatment of breast cancer play an important role in the aspect of reducing the mortality rate. At present, mammography with a high sensitivity and specificity is the main technique for breast cancer detection, but its limitation is an urgent problem to solve. On the one hand, the effect of early mammography is not ideal for women with dense breasts [3,4]. On the other hand, the ionizing radiation produced by mammography is potentially harmful to patients. Therefore, the mammography presents some inadequacies for the early screening for breast cancer. For this dilemma, ultrasonography (US), as an alternative means of diagnosing breast cancer, is able to compensate for the inadequacy of mammography [5]. US can distinguish tissues according to the differences of the acoustic impedance. It has the advantages of no pain, no trauma, a simple operation, no harm to people and a low price, such that has been widely used in clinics [6,7,8,9]. It is worth noting that the most common diagnostic ultrasound transducer operates at a frequency of 2.5 to 7.0 MHz [10]. Based on the above advantages, some teams have developed complete ultrasound computed tomography (USCT) systems.

In previous studies, the group of Duric and others, Karmanos Cancer Institute, Michigan, developed the first breast imaging device, based on the principle of ultrasound tomography for clinical evaluation [11]. The team reported on a SoftVue breast ultrasound imaging system in [12], and evaluated its performance. The team of Gemmeke studied the three-dimensional ultrasound CT imaging experimental device on the basis of the two-dimensional ultrasound CT experimental device [13]. The group of Michael Andre developed a clinical prototype of the computer volume ultrasound system (CVUS) [14]. The team of the Karlsruhe Institute of Technology successfully developed the KIT 3D USCT system and carried out continuous clinical trials [15]. DRAMI’NSKI S.A. developed the ultrasound tomography scanner model that is used for in vivo imaging analysis [16]. Professor Ming Yue Ding’s team developed a new USCT research system called Ultralucid with a high degree of scalability and flexibility [17]. Although linear arrays are commonly used for ultrasound imaging, most breast imaging systems use the ring array. Because the ring arrays not only cover the 360 degree range, but also have an approximately isotropic resolution [18,19].

The development of ultrasound imaging systems has achieved remarkable results by a number of research teams, but the use of different combinations of transducers and sound field analyses are still important to improve the image quality. In this paper, we addressed the multi-element synthetic aperture focusing technology (M-SAF) applied in ultrasound imaging using ring arrays. In [20,21], the concept of a synthetic aperture used for the high resolution ultrasonic imaging was introduced and focused on the research of synthetic apertures, respectively. Through the analysis of the sound field distribution of different array elements and through simulations and experiments, the best imaging results are obtained. This paper is organized as follows. Section 2 carries out the modeling and formula derivation of the ring array sound field. Section 3 not only carries out a sound beam and directivity simulation that is similar to the literature [22,23], but also simulates the data acquisition and image reconstruction in a k-Wave toolbox. In [24,25], the simulation of a numerical phantom and ultrasonic transducers is realized using k-Wave. Section 4 performs the experiments for the ultrasound imaging of the nylon ropes and breast model introduced in literature [26]. Finally, Section 5 concludes and discusses this work.

## 2. Methods

### 2.1. Imaging System

**Figure 1:**
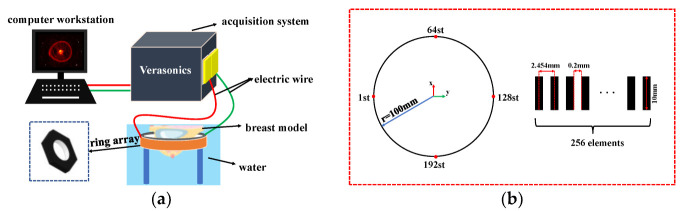
Schematic diagram of the ring array data acquisition system. (**a**) Schematic of the US imaging system based on the ring array. (**b**) Element parameters in the ring array.

A data acquisition system based on a ring array is Vantage-256 of Verasonics Inc. (Verasonics, a privately held company founded in 2001, is headquartered in Kirkland, Washington, DC, USA). Figure 1a shows the schematic diagram of the ring array data acquisition system. The hardware part is mainly composed of the computer workstation, the Verasonics acquisition system and the ring array. The detection depth of the transducer decreases with the increase of the center frequency. Therefore, in order to ensure that the ultrasonic transducer can detect any position of the breast and be within the working frequency range (2.5–7 MHz) of the diagnostic ultrasonic transducer, we have customized a 256 ring array with a center frequency of 3 MHz and a diameter of 200 mm. The ring array is specially customized, with 256 elements equally spaced within 360 degrees. As shown in Figure 1b, The radius of the ring array is 100 mm; the center distance of the adjacent elements is 2.454 mm; the spacing of the adjacent elements is 0.2 mm; the length of a single element is 10 mm, the center frequency of each element is 3 MHz; the transmission and reception bandwidth is about 60%, and 128 channels are used for the synchronous acquisition. The sampling resolution is 14 bits and the sampling rate is 11.9 MHz [27,28].

### 2.2. Wave Transmitting Method

**Figure 2:**
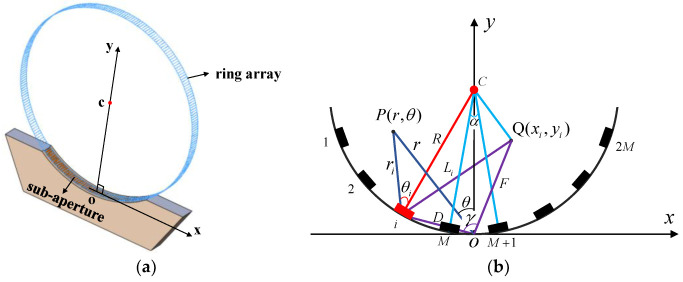
Schematic diagram of the sub-aperture in the ring array. (**a**) Three dimensional modeling of the ring array and the sub-aperture. (**b**) The Cartesian coordinate system on the *xoy* plane established for the sub-aperture.

Figure 2 is the schematic diagram of the ring array and the synthetic aperture with a concave acoustic radiation surface. The rectangular elements are uniformly distributed. The center of the ring array is set as the point *c*, the coordinate origin is set as point *o*, the *x* axis represents the tangent direction, and the *y* axis stands for the radial direction. The sound field distribution characteristics of the ring array are analyzed using the Cartesian coordinate system.

N represents the number of elements of the sub-aperture in the ring array. When N=1, the sound pressure at any point generated by the rectangular element with the center at the origin, can be obtained using Equation (1) in the xoy coordinate [22].

(1) $$p(r,\theta ,t)=\frac{p_{0}a}{r}\frac{\sin(\delta )}{\delta }e^{-j\delta }e^{j(\omega t-kr)}$$

where variable p(r,θ,t) represents the sound pressure of the coordinate point (r,θ) at t time. $p_{0}$ denotes the sound pressure amplitude of the sound source, and a stands for the element width. Notations ω, k and δ can be expressed as below:

(2) $$\left\{\begin{array}{l} \delta =\frac{1}{2}ka\sin\theta \\ \omega =2\pi f\\ k=\frac{1}{\lambda } \end{array}\right.$$

These are common symbols in the field of acoustics. Where ω represents the angular frequency, k denotes the wave number, f stands for the center frequency, and λ is the wavelength. When N>1, the value of N represents the even number of elements that are numbered from left to right (1,2,⋯,i,⋯,N), and N=2M, as shown in the Figure 2b. When the sound beam is focused on point Q, the sound pressure at any point is shown in Equation (3) in the far field, which can be obtained by calculating the sum of the sound pressure generated by all elements in the sub-aperture.

(3) $$p(r,\theta ,t)=\sum_{i=1}^{2M}p_{i}(r,\theta ,t)=\sum_{i=1}^{2M}\frac{p_{0}a}{r_{i}}\frac{\sin(\delta_{i})}{\delta_{i}}e^{-j\delta_{i}}e^{j(\omega t_{i}-kr_{i})}$$

(4) $$\delta_{i}=\frac{1}{2}ka\sin\theta_{i}$$

where $r_{i}$ represents the distance from the center point of the element i to the point P, and $t_{i}$ is the time required for the sound wave emitted from element i to point P. Parameter $\delta_{i}$ can be obtained by substituting parameter $\theta_{i}$ into Equation (4), where $\theta_{i}$ is the included angle between the vertical line passing through the center of the element i and the connecting line between the element and point P. The delay of each element is different, since the condition of the beam focusing is that the emission time of each array element is different, as shown in Equation (5).

(5) $$t_{i}=t-\Delta \tau_{i}$$

where t represents the time period from the first element to the current one. The variable $\Delta \tau_{i}$ is the relative delay between the element i and the transmitting element 1, which can be expressed through substitute Equations (7) and (8) into Equation (6).

(6) $$\Delta \tau_{i}=t-\frac{L_{i}-F}{c}$$

According to the cosine theorem, the distance $L_{i}$ from the element i to the focal point Q can be described by Equation (7).

(7) $$L_{i}=\sqrt{4R^{2}\sin^{2}[(i-1-\frac{M-1}{2})\alpha ]+F^{2}-2DF\cos\gamma }$$

where R is the radius of the curvature of the arc, α represents the center angle of the adjacent elements, D stands for the distance from the element i to the point o, and F denotes the length of the focus Q distance point o. In addition, angle γ needs to be obtained according to the geometric relationship of the ΔioQ.

(8) $$\gamma =\frac{\pi -(i-1-\frac{M-1}{2})\alpha }{2}-arc\tan\frac{x_{i}}{y_{i}}$$

where $x_{i}$ and $y_{i}$ are the coordinates of the focus point Q.

### 2.3. US Sequence Waves

**Figure 3:**
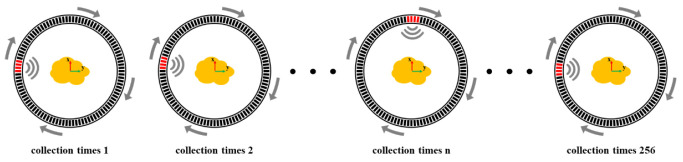
Schematic of the control mode of the receiving elements and the transmitting elements based on the M-SAF. The elements of the sub-aperture move an element clockwise in the ring array, as shown in the red arrays. M-SAF: multi-element synthetic aperture focusing.

A schematic of the ring-array-based control method is shown in Figure 3. All elements have the same properties and efficiency. The black parts of Figure 3 indicate the inactive elements, which are not involved in data acquisition. The red parts are the active elements during the data acquisition that are the sub-aperture mentioned below. By applying an excitation signal to the elements in the sub-aperture, the ultrasonic wave generated undergoes a variety of complex propagation processes, such as reflection, scattering and transmission, and is received by the same elements. So far, the receiving and transmitting process is completed. Moreover, the number of elements remains unchanged. Then, the sub-aperture moves the position of an element counterclockwise and repeats the above process 255 times. Finally, the acquired data are subjected to the echo signal extraction, the logarithmic compression and the sliding average.

## 3. Simulation and Results

### 3.1. Sound Field Beam Simulation

**Figure 4:**
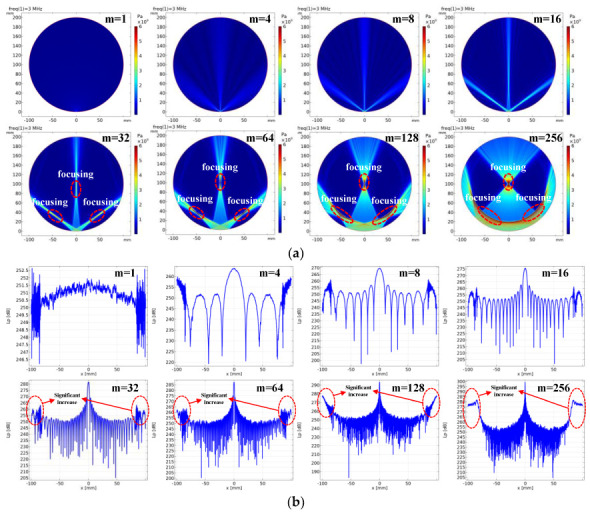
Acoustic field pattern and beam directivity pattern of the different number of active elements in the ring array (m = 1, 4, 8, 16, 32, 64, 128, 256). (**a**) Acoustic field distribution results formed by the different number of active elements. (**b**) Calculation results of the beam direction formed by the different number of active elements in the ring array.

Since the diameter of the ring array is much larger than the length of a single element, the acoustic field can simplify the plane of the two-dimensional acoustic field. By analyzing the acoustic field distribution and the beam directivity, the number of the sub-aperture’s optimal elements can be determined, thereby improving the image quality. Based on COMSOL, the two-dimensional frequency domain simulation environment, a 1024 ring array with a 200 mm diameter is constructed. The elements are equally spaced in the 360-degrees range, and the angle is 0.3516 degree and the center distance is 0.6136 mm between the adjacent elements. The interior of the ring array is set as the water area, with the sound velocity of 1540 m/s and the density of 1000 Kg/m^3^. The elements are replaced by a point source to ensure that the wave emitted by each element is spherical, and the radiation boundary is set as the plane wave radiation. In order to meet the accuracy requirements, the mesh is divided into λ/5 (Selection of λ/5 refers to the COMSOL blog case: *Using Simulation to Study Ultrasound Focusing for Clinical Applications*). The sparse direct solver is selected for the calculation, which can realize the real and complex operations and has a complete linear system matrix. The maximum frequency of the solver is set to 3 MHz. As shown in Figure 4, 1024 elements are evenly distributed on the ring array, and only the fixed elements are activated for transmission each time. In this paper, we select a certain number of transmitting elements (m = 1, 4, 8, 16, 32, 64, 128, 256) to calculate the sound field distribution and the beam direction in the ring array.

As shown in Figure 4a,b, the optimal number of elements is 16, which is characterized by no beam focusing, a narrow main lobe and a low side lobe. If the number of array elements is less than 16, the sound pressure is weak in the ring array, and the main lobe is wide in the directivity diagram, which is easy to lead to a low imaging quality. If the number of elements is more than 16, the beam obviously focuses on the non-round center, and the sound pressure level is significantly enhanced at both ends of the directivity diagram, which has a bad impact on the imaging results, resulting in a low imaging quality.

### 3.2. Point Target Simulation

**Figure 5:**
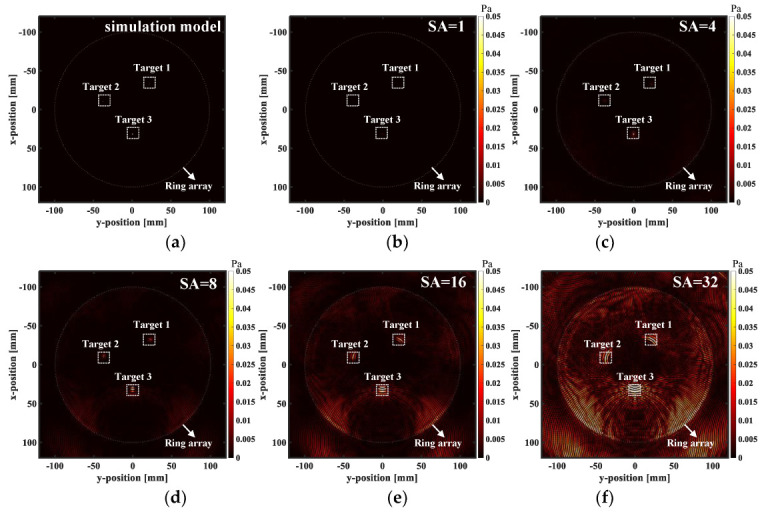

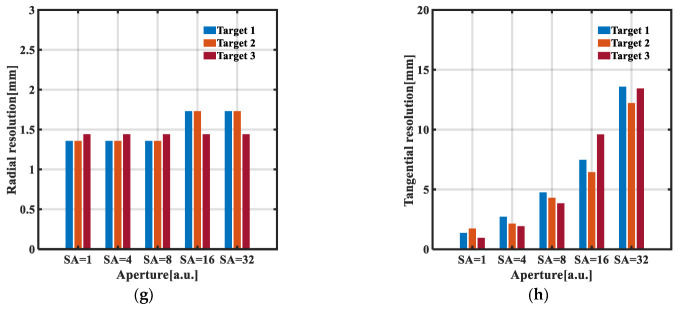
Simulation of the point target with a different aperture (SA = 1, 4, 8, 16, 32). (**a**) Three different targets are built in the simulation model, Target 1 with a radius of 0.25 mm, Target 2 with a radius of 0.5 mm and Target 3 with a radius of 0.75 mm. (**b**–**f**) The reconstructed US image using the SA method.(**g**,**h**) Bar chart comparison of the spatial resolution of the three different targets. SA: synthetic aperture.

As an important basis for the imaging quality evaluation, the spatial resolution can be expressed by the full width at half maximum (FWHM). Figure 5a shows that three targets are established in the 256 ring array with a diameter of 200 mm, based on the k-Wave simulation environment. The target is placed in a uniform medium with a sound velocity of 1500 m/s and a density of 1000 Kg/m^3^. Target 1, as the smallest unit that can be divided in the figure, tends to be infinitesimal and has a radius of 0.25 mm. Therefore, Target 1 is approximated as a point source. Target 2 and Target 3 are the aggregation of the multi-point sources, and their radii are 0.5 mm and 0.75 mm, respectively. The transmission center frequency of each array element is 3 MHz, and the generated signal excitation is a modulation wave of five cycles. The center distance of the array elements is 0.614 mm, and the angle between the adjacent elements is 0.35°.

The signal is collected through the M-SAF control mode. In other words, the number of transmitting elements is attached. Following each transmission, one element moves backward to the end and turns. The receiving elements and the transmitting elements are the same group of elements. The model is used to synthesize the aperture reflection imaging in MATLAB R2019a. As shown in Figure 5b–f, the sound pressure at three different targets increases with the increase of the aperture size, and the background noise also increases. If the aperture size is one element or four elements (SA = 1 or SA = 4), the target is not distorted in the reconstruction area, and the tangential dimension is close to the radial dimension. If the aperture size is eight elements (SA = 8), the target begins to produce distortion in the reconstructed area, and the tangential size is larger than the radial size. If the aperture size is 16 and 32 elements (SA = 16, 32), the distortion of the reconstructed area is more serious. In order to make the expression more intuitive, the sound pressure amplitude is detected in the tangential and radial directions of the target, respectively, and the FWHM in the radial and tangential directions are calculated. The results are shown in Figure 5g,h. The FWHM of the three targets has significantly different variation rules in the radial and tangential directions. Figure 5g shows the FWHM of the three targets in the radial direction. The FWHM of Target 1 and Target 2 is 1.36–1.73 mm, and the FWHM of Target 3 is 1.44 mm. The FWHM floats gently and remains around 1.5 mm. Figure 5h shows the FWHM of the three targets in the tangential direction. The FWHM of Target 1 is 1.36–13.58 mm; the FWHM of Target 2 is 1.73–12.22 mm, and the FWHM of Target 3 is 0.96–13.44 mm. The FWHM of the three targets shows an increasing trend and gradually deviates from the tangential size of the target. Considering the target distortion and the sound pressure amplitude, four elements can be considered as a suitable aperture size, which is consistent with the theory.

## 4. Experiments and Results

### 4.1. US Imaging of the Nylon Rope

**Figure 6:**
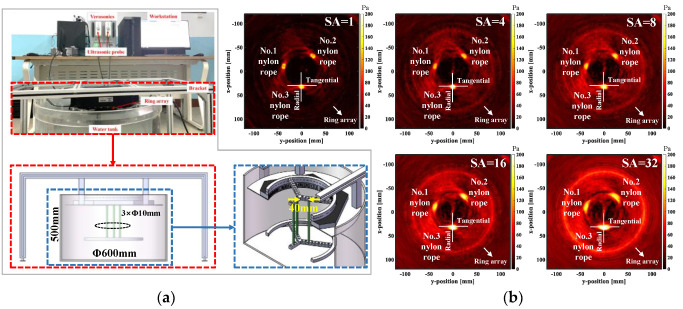

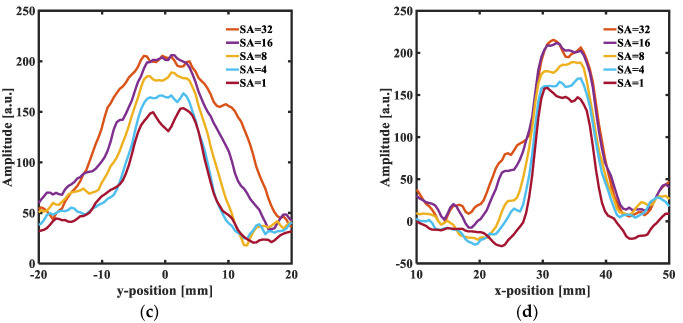
US imaging of the nylon rope based on M-SAF (SA = 1, 4, 8, 16, 32). (**a**) US imaging system and partial three-dimensional modeling. (**b**) Reconstruction results of the nylon rope ultrasonic imaging. (**c**) Radial sound pressure amplitude detection of the nylon rope in the reconstructed image. (**d**) Amplitude detection of the tangential sound pressure value of the nylon rope in the reconstructed image. M-SAF: multi-element synthetic aperture focusing; US: Ultrasonic imaging; SA: synthetic aperture.

As the acoustic impedance of the nylon rope is slightly different from that of water, it can be used as a preliminary imaging study for tumors. For example, the nylon rope was used as the test object in the experimental section of the literature [29,30,31]. So, three nylon ropes with a 10 mm diameter and consistent acoustic characteristics, are selected, and the cross section of the nylon rope is circular in this paper. Both ends of the nylon ropes are respectively fixed on the Y-shaped frame in two rings, and the nylon ropes are kept parallel. The sticks are distributed at equal angles in the ring, and the adjacent angles at 120 degrees. One end of the fixed nylon rope is placed in the water tank and the other end is placed on the upper surface of the ring array. At the same time, the three nylon ropes vertically pass through the cross section of the ring array and are distributed inside the ring array at an equal angle. Each nylon rope is 40 mm away from the center of the ring array, as shown in Figure 6a.

The US imaging experiment of the nylon rope is carried out using M-SAF. Figure 6a shows the US imaging system in the nylon rope experiments and the three-dimensional modeling of the local device. Figure 6b shows the US imaging of the aperture with a different number of elements in the nylon rope experiment. The amplitude of the sound pressure and the background noise in the reconstructed US imaging of the nylon rope increases with the increase of the SA.

As the aperture size is one and four elements (SA = 1, 4), it has the characteristics of a high contrast and a low background noise. The shape of the nylon rope is close to circular in the reconstructed US imaging, which is consistent with the measured model. When the SA increases from 8 to 32 (SA = 8, 16, 32), the background noise increases significantly, and the shape of the nylon rope gradually tends to ellipse in the reconstructed US image, which indicates that the imaging quality decreases. Taking nylon rope No. 3 in the reconstructed image as an example, the amplitude of the sound pressure is detected along the radial and tangential directions in the reconstructed US image, and the change in the nylon rope size is observed in the reconstructed image, as shown in Figure 6c,d. Table 1 shows the nylon rope reconstruction dimensions and errors, which hare used in a further quantitative analysis of the results in Figure 6c,d. Figure 6c shows the tangential size of nylon rope No. 3 in the reconstructed image. When the SA = 1, the dimension of the reconstructed nylon rope is 9.32 mm, and the reconstruction error is 6.8%. When the SA = 4, the dimension of the reconstructed nylon rope is 10.81 mm, and the reconstruction error is 8.1%. When the SA increases from 8 to 32, the dimension of the reconstructed nylon rope is 15.36–24.00 mm, and the reconstruction error is 53.6–140.0%, which deviates significantly from the actual size. Figure 6d shows the radial size of nylon rope No. 3 in the reconstructed image. When the SA = 1, the dimension of reconstructed nylon rope is 9.12 mm, and the reconstruction error is 8.8%. When the SA = 4, the dimension of the reconstructed nylon rope is 10.56 mm, and the reconstruction error is 5.6%. When the SA increases from 8 to 32, the dimension of the reconstructed nylon rope is 12.00–15.84 mm, and the reconstruction error is 20.0–58.4%. It deviates significantly from the actual size, but the deviation is much less than that shown in Figure 6c. The result indicates that the tangential size of the measured object is easier to deviate from the actual situation in the process of the ultrasonic imaging, based on M-SAF. Considering the weak signal intensity of the single element and the distortion degree of the reconstructed image, it is reasonable to select the synthetic aperture with four elements.

### 4.2. US Imaging of the Breast Model

For the purpose of studying breast imaging, a breast model with similar acoustic characteristics of an actual breast, is customized. The breast model was produced by Phantom Shenzhen Well Come Technology co. LTD, China (The company specializes in the sales and customization of medical testing instruments and models). The model, with a size of 155 mm × 135 mm × 80 mm, is made of proprietary gel, similar to human tissue and is wrapped around a layer of skin-like elastic membrane. A tumor with a diameter of 25 mm is embedded in the center of the breast model, and is made of polyurethane rubber. The sound velocity is 1520 m/s, the density is 1032 Kg/m^3^ and the operating frequency range is 2–4 MHz.

**Figure 7:**
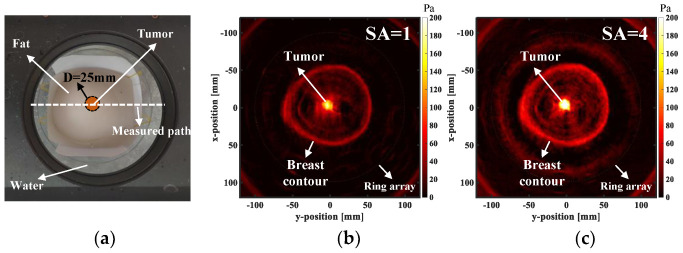

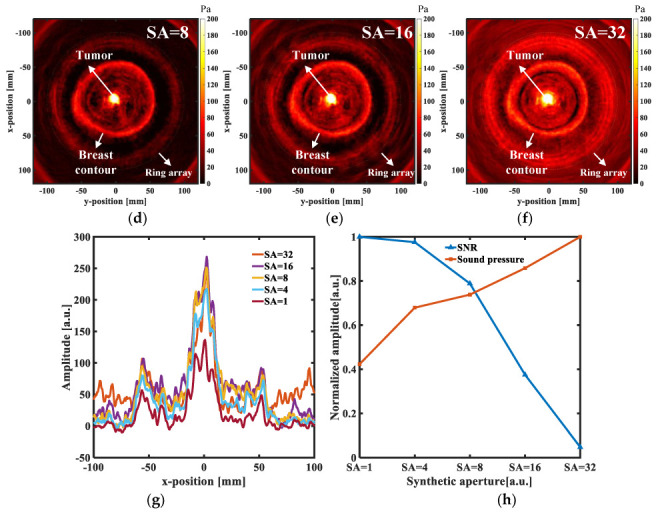
US imaging of the breast model, based on M-SAF (SA = 1, 4, 8, 16, 32). (**a**) The breast model is located in the center of the ring array, and there is a tumor in the center of the breast model. The diameter of the ring array is 200 mm, the size of the breast model is 155 mm × 135 mm × 80 mm and the diameter of the tumor is 25 mm. (**b**–**f**) Reconstruction results of the breast model US imaging. (**g**) Variation of the sound pressure amplitude on the measured path. (**h**) Variation of the normalized sound pressure amplitude and the SNR with SA. M-SAF, multi-element synthetic aperture focusing; US, ultrasonography; SNR, signal-to-noise ratio; SA, synthetic aperture.

The breast model is fixed to the center of the ring array, and the top point of the breast model is 4 cm away from the upper surface of the ring array, as shown in Figure 7a. The US imaging of the breast model is performed, based on M-SAF with different elements (SA = 1, 4, 8, 16, 32). Then, the influence of the aperture size on the imaging quality is further compared by detecting the sound pressure amplitude of the measured path in Figure 7a. The results, as shown in Figure 7b−f, are that the sound pressure amplitude and background noise in the reconstruction images increase with the increase of the aperture size, and the signal-to-noise ratio (SNR) decreases with the increase of the aperture size. The SNR values are shown in Table 2. When SA = 1 or SA = 4, the amplitude of the sound pressure inside and outside of the breast contour changes greatly, which means that the edge definition is high, and the reconstructed image quality is high. It can intuitively present the shape and location distribution of the breast model and tumor, and can provide effective and richly detailed information for further analysis. When the SA increases from 8 to 32, the variation degree of the sound pressure amplitude on the inside and outside of the breast contour decreases, due to the increase of the background noise. It means that the quality of the reconstructed image decreases, and the detail information is affected by the background noise. Therefore, we should comprehensively consider the relationship between the SNR and the sound pressure amplitude when selecting the appropriate subarray elements, so as to ensure the quality requirements of the reconstructed image. As shown in the curve in Figure 7d, the sound pressure amplitude increases with the increase of the SA, and the SNR decreases with the increase of the SA. Considering the influence of the sound pressure amplitude and the SNR on the imaging quality, a SA = 4 is the most suitable. The sound pressure amplitude and the SNR maintain a high level, and the imaging quality is the highest, which is consistent with the theory.

The medium cannot determine the sound pressure. Because the sound pressure is not only related to the sound velocity and density, but also related to the sound intensity. The sound intensity is affected by the propagation distance and the attenuation coefficient. In this paper, the acoustic characteristics of the nylon rope and the breast model are different. Therefore, their sound pressure amplitude detection results are slightly different. The brightness of color in the ultrasonic imaging reflects the change of sound pressure value. The color brightness is proportional to the sound pressure value. For example, the position with a higher value in Figure 6c–d, is the brightest position on the measured path in Figure 6b. Figure 7 also has a similar correlation.

The optimal sub-aperture theory is verified in the 1024 ring array and 256 ring array. Compared with the 256 ring array, the 1024 ring array can be divided into more sub-apertures for research, which can be achieved in COMSOL, and the results are more convincing. Although the experiment is limited by the experimental conditions in which only the imaging of the 256 ring array is allowed, the resolution evaluation, the nylon rope and the breast model imaging experiments can also verify the existence of the theory in the 256 ring array.

## 5. Conclusions

In summary, the paper introduces the application of M-SAF in the US imaging of a ring array. In addition, the imaging of the breast cross-section is analyzed from the angle of the sound field generated by the elements. The feasibility is validated by a breast model with real breast acoustic characteristics. Through the establishment of the 1024 ring array in COMSOL, the sound field beam simulation and directivity simulation are carried out. The results show that the optimal number of elements in the 1024 ring array is 16. Specifically, the narrower the main lobe is, the higher the sound pressure value and the better the directivity will be. If the number of elements is less than 16, the main lobe is wider and the side lobe is higher than in the directivity diagram. If the number of elements is more than 16, the sound field beam produces multi-point focusing, and the sound pressure level increases significantly at both ends. Once the spatial resolution, reconstruction error, sound pressure intensity and signal-to-noise ratio have been comprehensively considered, SA = 4 has the highest imaging quality in the simulation and in the experiment of the 256 ring array. The radial resolutions of Targets 1, 2 and 3 are 1.36 mm, 1.36 mm and 1.44 mm, respectively, and the tangential resolution is 2.72 mm, 2.14 mm and 1.92 mm. According to the sound pressure amplitude of the nylon rope, the radial reconstruction error is 5.6%, and the tangential reconstruction error is 8.1%. In the breast model reconstruction image, the normalized sound pressure amplitude is 0.68, and the SNR is 17.14 dB. If SA < 4, the normalized sound pressure amplitude is the smallest (0.43), which leads to low imaging quality. If SA > 4, the nylon rope distortion, the nylon rope reconstruction error and the background noise gradually increase, while the SNR decreases, which results in a low imaging quality. It is worth noting that the difference between the acoustic characteristics of the tested object and the measurement environment may lead to slightly different results in the mainly experimental stage. For example, the different acoustic impedance of the nylon rope will cause a slight change in the reconstruction error. The number of elements is greater than 256, which may lead to the optimal number of elements not equal to four.

At present, the imaging system has some limitations. Due to the low number of elements and the large diameter, the element pitch of the ring array is large, which leads to a low quality of the reconstructed image. To solve the above problems, one method is to directly purchase the 1024 ring array, which can directly obtain the RF data without expanding the amount of data through data processing. This method can realize a high-resolution imaging to a large extent, but the hardware cost is too high. For example, the price of the Doppler 256 × 32 piezoelectric cylindrical array is more than 1 million CNY. Another method is to recover the channel data of the 256 ring array, so as to achieve the data volume of the 1024 ring array. On this basis, US imaging based on M-SAF is carried out. Although the data recovered by this method are not all original data, its cost is low. At the same time, the compressed sensing or deep learning methods may provide us with a new idea to improve the imaging quality. Because these methods have been applied in other fields [32,33,34].

## Figures and Tables

**Table 1:** Reconstruction of the dimensions and errors of the nylon rope.

Direction	Index	SA = 1	SA = 4	SA = 8	SA = 16	SA = 32
y-position	Dimension	9.32 mm	10.81 mm	15.36 mm	19.20 mm	24.00 mm
	Error	6.8%	8.1%	53.6%	92.0%	140.0%
x-position	Dimension	9.12 mm	10.56 mm	12.00 mm	12.96 mm	15.84 mm
	Error	8.8%	5.6%	20.0%	29.6%	58.4%

**Table 2:** SNR of the US imaging based on the different aperture sizes.

Types	SA = 1	SA = 4	SA = 8	SA = 16	SA = 32
SNR	17.57 dB	17.14 dB	13.85 dB	6.58 dB	0.84 dB
